# Giant subpatellar synovial osteochondromatosis complicated by osteoarthritis of the knee: A case report

**DOI:** 10.1097/MD.0000000000050834

**Published:** 2026-09-25

**Authors:** Chun Zhang, Jiaxin Fu, Zhiqing Chen, Jinxin Zheng, Yancheng Xiu, Shuzhang Guo

**Affiliations:** a Department of Orthopedics, The Third Central Hospital of Tianjin, Tianjin, China; b Department of Orthopedics, Tianjin Key Laboratory of Extracorporeal Life Support for Critical Diseases, Tianjin, China; c Department of Orthopedics, Artificial Cell Engineering Technology Research Center, Tianjin, China; d Department of Orthopedics, Tianjin Institute of Hepatobiliary Disease, Tianjin, China.

**Keywords:** osteoarthritis of the knee, subpatellar mass, synovial osteochondromatosis

## Abstract

**Rationale:**

Synovial osteochondromatosis (SOC) is a rare proliferative disorder of the synovium that most commonly affects the knee and is characterized by the formation of multiple cartilaginous nodules or intra-articular loose bodies. Subpatellar SOC is extremely rare because only a small amount of synovial tissue is present in the deep infrapatellar bursa. Prolonged mechanical irritation caused by subpatellar SOC may contribute to progressive cartilage damage and osteoarthritis. We report a patient with giant subpatellar SOC accompanied by severe knee osteoarthritis who underwent mass excision combined with total knee arthroplasty, a treatment approach that has rarely been described.

**Patient concerns:**

A previously healthy 71-year-old woman was admitted to our institution in March 2025 with a 2-year history of progressive pain and swelling in the left knee. Her symptoms had worsened during the preceding month, resulting in marked restriction of left-knee movement and substantial impairment of daily activities.

**Diagnoses:**

The patient was diagnosed with giant subpatellar SOC accompanied by severe osteoarthritis of the knee.

**Interventions:**

Examination revealed severe destruction of the left-knee articular cartilage, and the associated pain substantially impaired the patient’s daily activities. Because conservative treatment had failed to relieve her symptoms, she underwent excision of the giant subpatellar lesion combined with left total knee arthroplasty.

**Outcomes:**

Histopathological examination of the resected mass confirmed the diagnosis of giant subpatellar SOC. After surgery, the patient experienced substantial relief of left-knee pain and was able to walk with the assistance of a walker within 48 hours. At the latest follow-up, she reported no pain or gait impairment, and no clinical or radiological evidence of recurrence was observed at the 3-month follow-up.

**Lessons:**

SOC may arise in the subpatellar region despite the limited amount of synovial tissue and may occasionally develop into a large mass. Although this presentation is rare, SOC should be considered in the differential diagnosis of a calcified subpatellar mass to reduce the risk of misdiagnosis. Complete lesion excision combined with total knee arthroplasty may be an appropriate treatment option for patients with giant subpatellar SOC and end-stage knee osteoarthritis.

## 1. Introduction

Synovial osteochondromatosis (SOC), also known as synovial chondrometaplasia, is a proliferative disorder of the synovium characterized by cartilaginous metaplasia of subsynovial connective tissue, which may subsequently undergo calcification and form cartilaginous nodules or loose bodies.^[1]^ SOC most commonly affects the knee, accounting for approximately 50% to 60% of cases, followed by the hip, whereas involvement of the upper-extremity joints is uncommon.^[2]^ It typically presents as multiple scattered intra-articular nodules, although extra-articular involvement of bursae, tendons, or tendon sheaths may occasionally occur.^[3]^ The clinical presentation of knee SOC is nonspecific and may overlap with that of osteoarthritis, with patients commonly reporting chronic pain, swelling, reduced range of motion, crepitus, or mechanical locking.^[4,5]^

Subpatellar SOC is extremely rare and may extend into the infrapatellar fat pad, where it can gradually develop into a large mass. After extensive calcification, the lesion may mimic patellar duplication on imaging and consequently lead to misdiagnosis.^[6]^ Subpatellar SOC may additionally present as a palpable infrapatellar mass accompanied by progressive knee pain and restricted motion.^[6]^ Diagnosis is based on imaging assessment and histopathological confirmation.

## 2. Case presentation

A 71-year-old woman developed left knee pain without an identifiable precipitating factor 2 years before presentation. The pain was initially tolerable and was managed conservatively with symptomatic analgesic treatment. Her symptoms gradually worsened despite conservative treatment. During the month before admission, the left knee pain markedly worsened and was accompanied by restricted range of motion; because of the pain, she could walk only approximately 20 m. She presented to our hospital in March 2025. She had no relevant medical history or family history of hereditary disease.

Physical examination revealed swelling of the left knee with mild valgus deformity. The overlying skin was intact, with no erythema. A firm, poorly defined, minimally mobile mass measuring approximately 4 × 5 cm was palpable beneath the left patella. The mass was non-tender, with no local warmth or visible vascular abnormalities. Tenderness was present along both the medial and lateral joint lines. The range of motion was restricted to 20° to 90°, and the patellar grind test was positive. Laboratory examination showed a serum potassium level of 3.33 mmol/L (reference range, 3.35–5.50 mmol/L); the remaining laboratory findings were unremarkable. Plain radiographs revealed an irregular calcified lesion beneath the left patella, together with osteophyte formation and joint-space narrowing (Fig. 1A, B). Magnetic resonance imaging revealed a heterogeneous-signal mass beneath the patella, chondral damage with adjacent bone marrow edema and cystic changes, a grade III tear of the lateral meniscus, and joint effusion (Fig. 2A–C).

**Figure 1.**
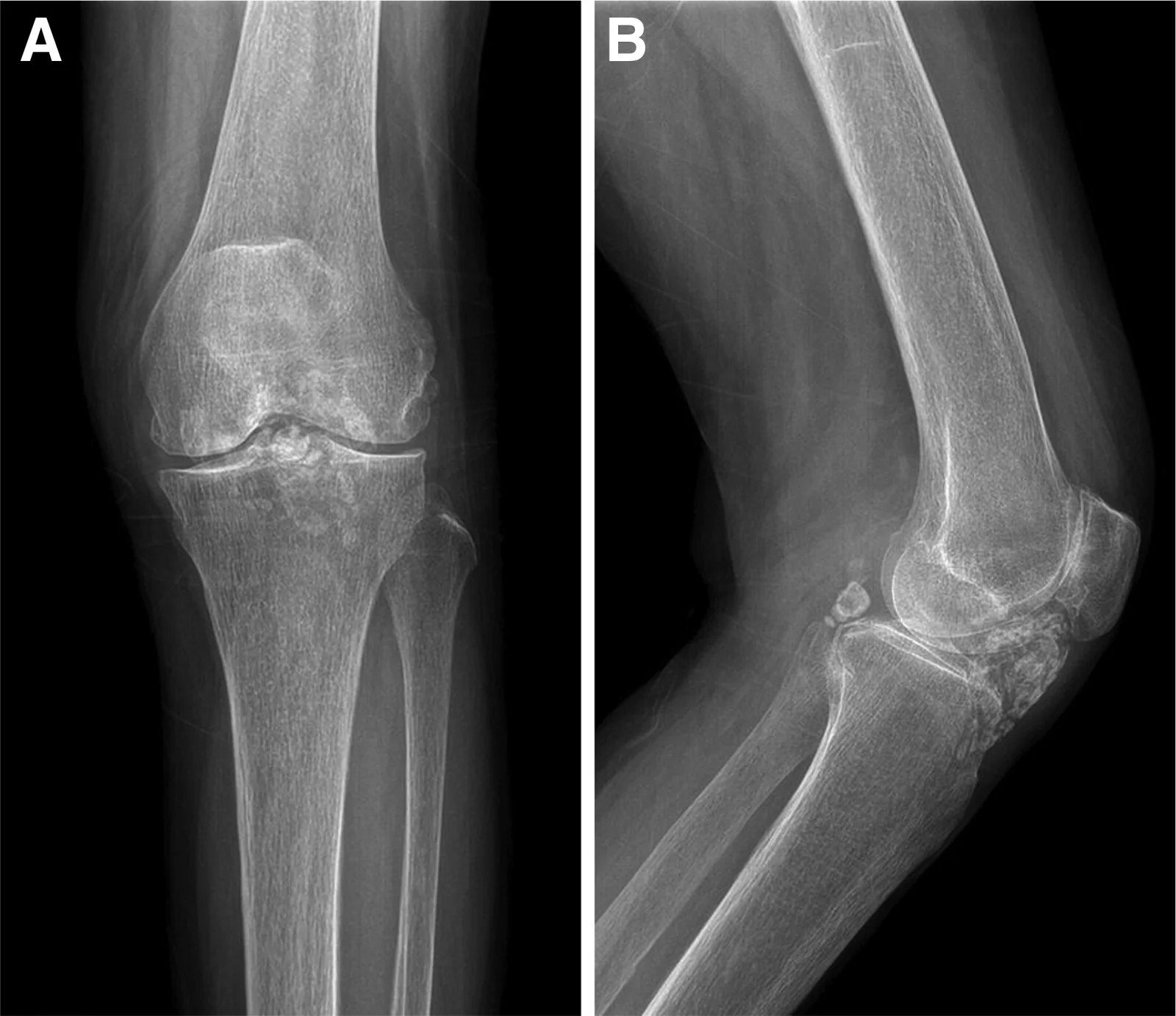
(A) X-ray: Osteophyte formation and narrowed joint space of the knee joint. (B) X-ray: An irregular high-density shadow beneath the patella of the left knee.

**Figure 2.**
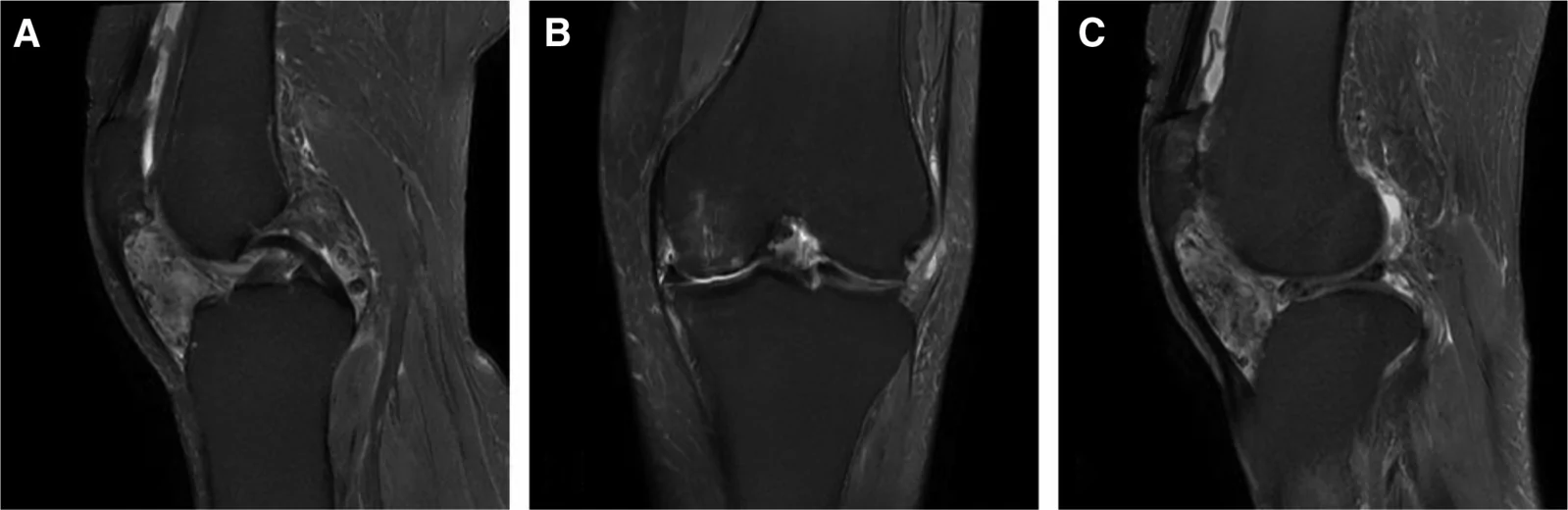
(A) MRI (sagittal plane): a mixed signal mass beneath the patella. (B) MRI (coronal plane): cartilage softening with surrounding bone marrow edema, cyst formation. (C) MRI (sagittal plane): Grade III lateral meniscus tear, and joint effusion. MRI = magnetic resonance imaging.

Given the severe pain, functional impairment, advanced osteoarthritis, and failure of conservative treatment, excision of the subpatellar mass combined with left total knee arthroplasty was planned. During surgery, a standard anterior midline incision was made, and severe articular cartilage destruction was observed. After flexion of the knee and eversion of the patella, a round, firm mass measuring approximately 4 × 5 cm was identified in the infrapatellar region. The lesion occupied the infrapatellar fat pad and was adherent to the surrounding tissues. It was carefully dissected from the adjacent structures and completely excised for histopathological examination (Fig. 3A–C). Total knee arthroplasty was subsequently performed because of the severe articular cartilage destruction (Fig. 4A, B). The wound was closed in layers, and the estimated intraoperative blood loss was 50 mL. Histopathological examination of the resected lesion revealed proliferative bone, cartilage, and fibrous connective tissue. Immunohistochemical staining was positive for S-100 and desmin, and the Ki-67 proliferation index was <1% (Fig. 5A–F).

**Figure 3.**
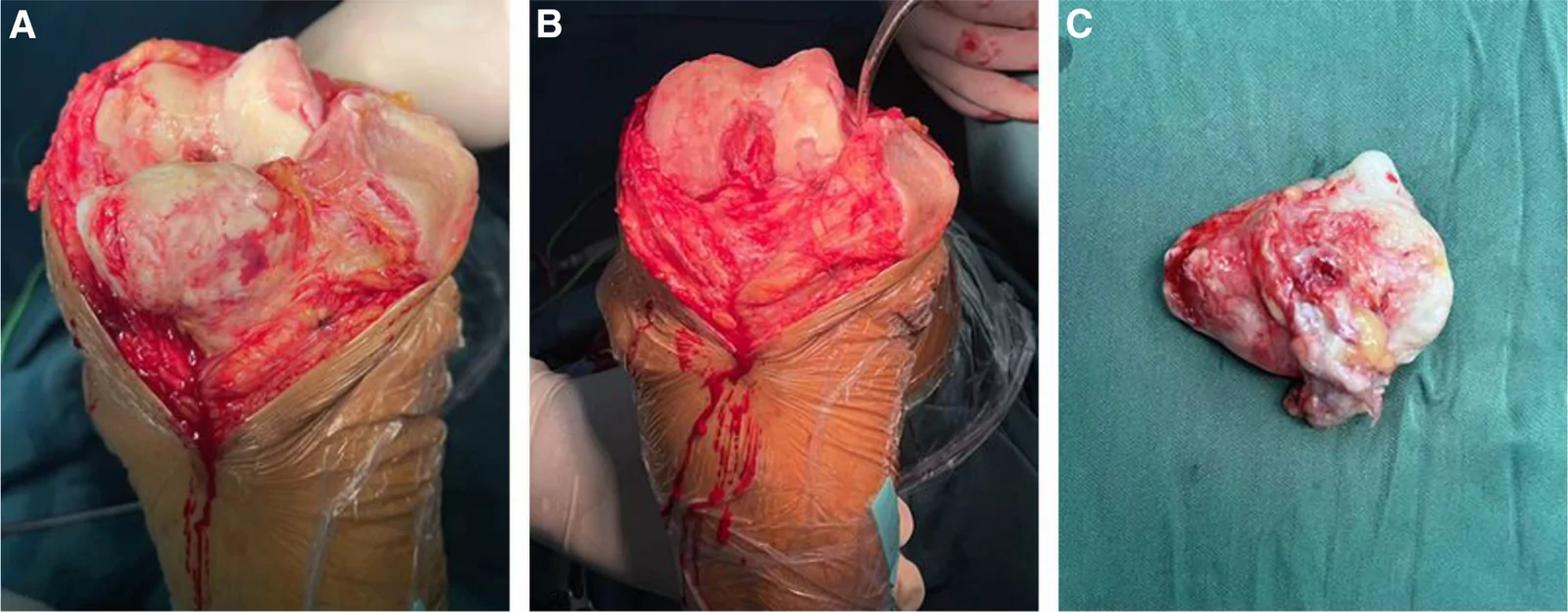
(A) The subpatellar mass shown during the operation. (B) Severe injury to the soft cartilage of the medial and lateral hoses of the femur (grade IV). (C) Synovial chondromatosis resected during the operation.

**Figure 4.**
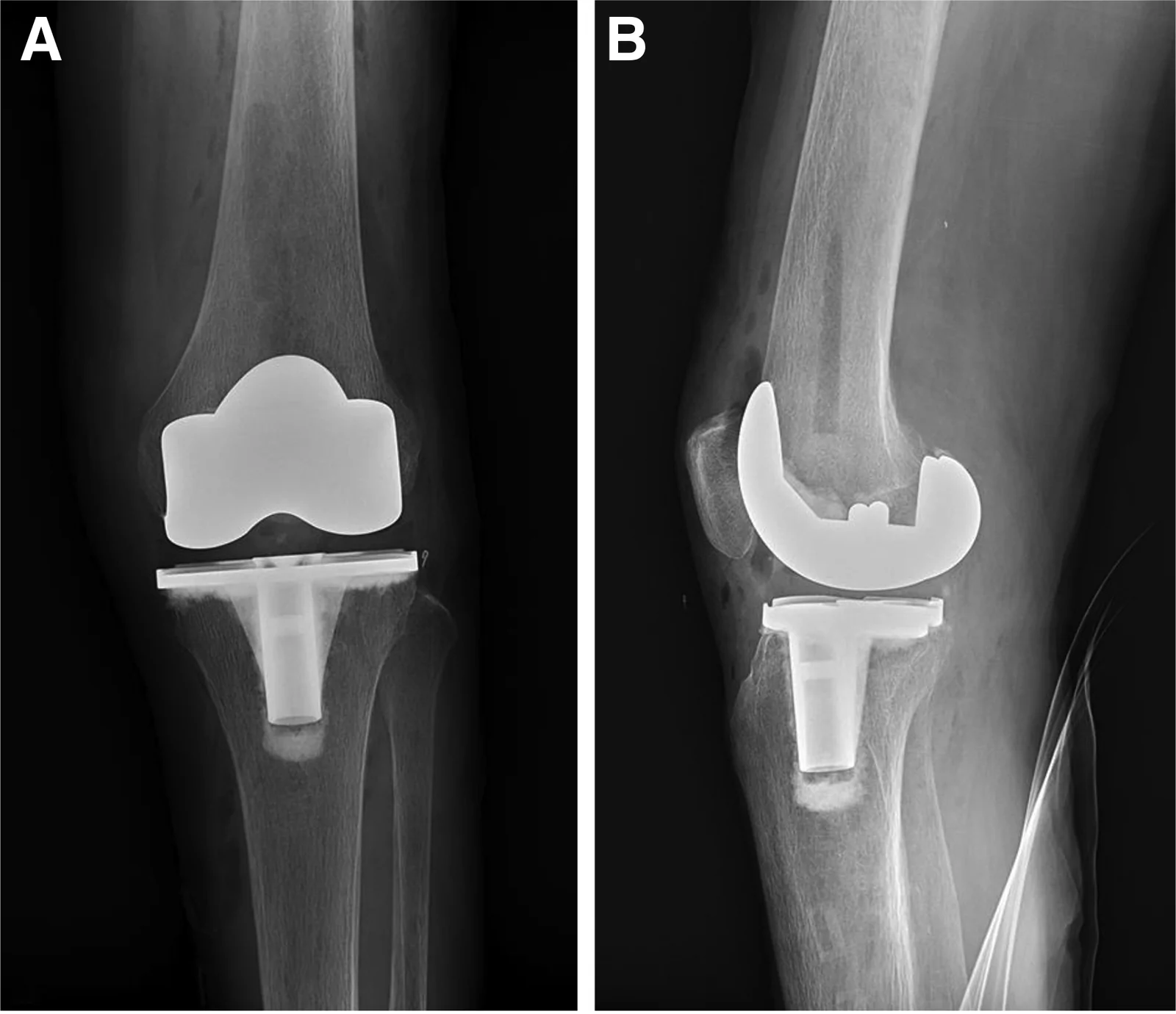
(A, B) Posteroanterior x-ray film of the keen of total knee arthroplasty.

**Figure 5.**
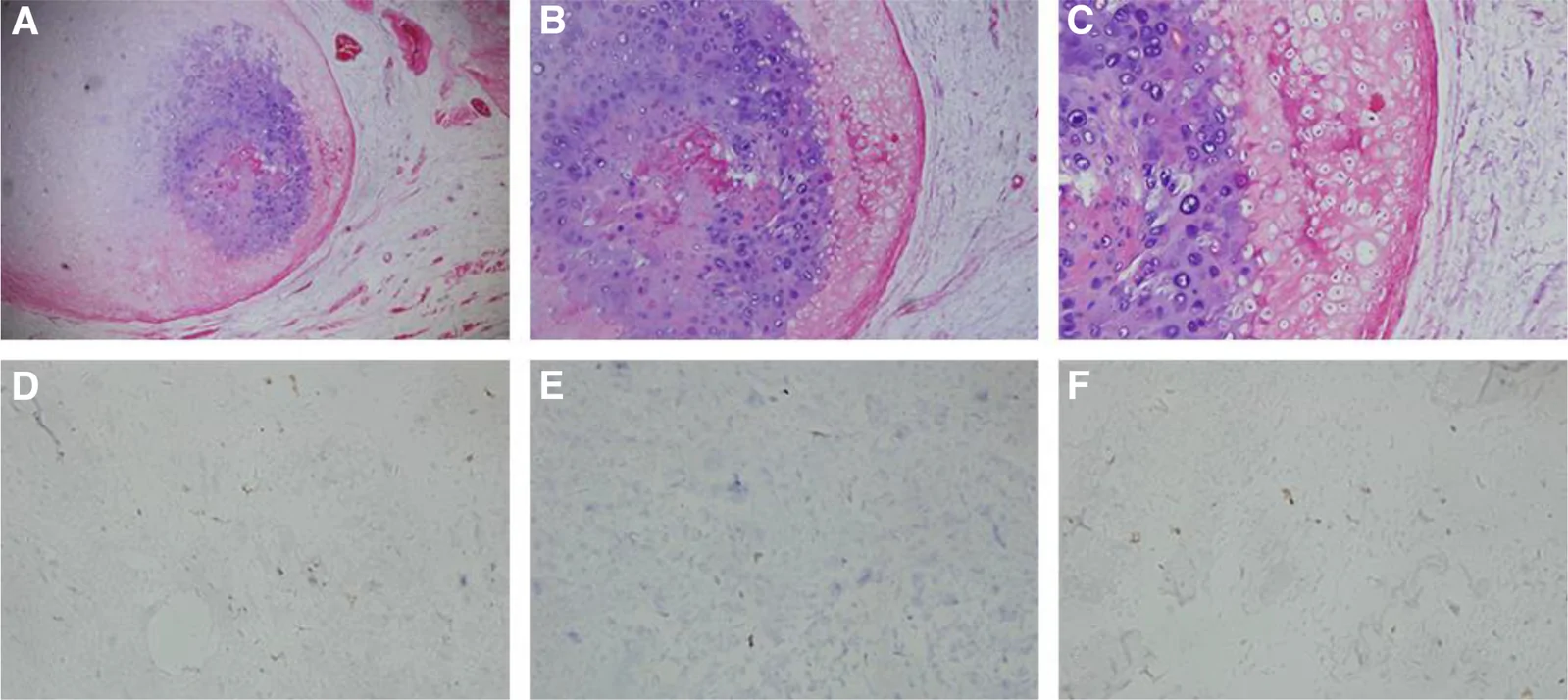
Pathologic histology of tumor specimens. (A) Microphotography showing characteristic nests of tumor cells (H&E, original magnification 40). (B) Microphotography showing characteristic nests of tumor cells (H&E, original magnification 100). (C) Microphotography showing characteristic nests of tumor cells (H&E, original magnification 200). (D) Immunohistochemistry of the lesion showed CD34 positive staining. (E) Immunohistochemistry of the lesion showed S100 positive staining. (F) Ki-67 immunostaining shows <1% Ki-67 positive cells.

The patient was able to stand and walk with the assistance of a walker within 48 hours after surgery. At the 1-month follow-up, her left knee pain had substantially improved. Physical examination showed no local erythema, swelling, or tenderness, and the patient was able to walk without restriction. No postoperative complications or clinical evidence of recurrence were observed.

## 3. Discussion

Subpatellar SOC is uncommon, and the clinical significance of the present case lies in the coexistence of a giant infrapatellar lesion and severe degenerative osteoarthritis requiring joint arthroplasty. The progressive knee pain, palpable 4 × 5 cm infrapatellar mass, restricted range of motion, calcified lesion beneath the patella, and additional loose bodies in the posterior knee raised suspicion of SOC. Magnetic resonance imaging (MRI) further defined the extent of the lesion and demonstrated associated cartilage damage, meniscal injury, bone marrow edema, and joint effusion. Histopathological examination showing proliferative bone, cartilage, and fibrous connective tissue confirmed the diagnosis.

The imaging appearance of SOC varies according to the extent of cartilage mineralization. Plain radiographs may identify calcified nodules or loose bodies, whereas computed tomography and MRI can more accurately define lesion location, soft-tissue extent, and relationships with adjacent structures.^[7-10]^ In the present case, the dominant mass-like configuration beneath the patella was atypical and could resemble an accessory or duplicated patella or another mineralized periarticular lesion.^[6]^ The absence of destructive changes in the adjacent bone supported a benign process, although definitive diagnosis required histopathological examination.^[11]^

Surgical management should be individualized according to lesion size, accessibility, synovial involvement, and the degree of articular degeneration. Arthroscopic loose-body removal with synovectomy is commonly used for accessible intra-articular disease, while open excision can facilitate complete removal of a large or firmly adherent mass.^[12-14]^ In this patient, the lesion occupied the infrapatellar fat pad and adhered to the surrounding tissues, supporting open excision. The indication for total knee arthroplasty was the severe articular cartilage destruction and disabling osteoarthritis; excision of the SOC was performed concurrently. Arthroplasty has previously been used in patients with SOC and advanced degenerative joint disease, although residual or recurrent lesions may affect prosthetic function and should be considered during follow-up.^[15,16]^ At the 3-month follow-up, the patient showed substantial pain relief and improved walking ability, with no evidence of recurrence identified.

This case has several limitations. Previous imaging and longitudinal clinical records were unavailable, precluding determination of whether the osteoarthritis primarily reflected age-related degeneration, secondary cartilage damage caused by long-standing SOC, or the combined effects of both processes. In addition, the 3-month follow-up period was insufficient to assess long-term recurrence or prosthetic outcomes. Continued clinical and imaging surveillance is warranted.

## 4. Conclusion

Giant subpatellar SOC should be considered in the differential diagnosis of a calcified infrapatellar mass accompanied by knee pain, restricted motion, and degenerative changes. In patients with a large lesion and end-stage osteoarthritis, complete mass excision combined with total knee arthroplasty may represent an individualized surgical option.

## Author contributions

**Conceptualization:** Zhiqing Chen.

**Data curation:** Chun Zhang, Shuzhang Guo.

**Investigation:** Chun Zhang.

**Formal analysis:** Shuzhang Guo.

**Funding acquisition:** Jiaxin Fu.

**Project administration:** Jiaxin Fu.

**Supervision:** Zhiqing Chen.

**Validation:** Zhiqing Chen, Jinxin Zheng.

**Visualization:** Jinxin Zheng.

**Writing – original draft:** Yancheng Xiu.

**Writing – review & editing:** Yancheng Xiu.
